# Antimicrobial food packaging: potential and pitfalls

**DOI:** 10.3389/fmicb.2015.00611

**Published:** 2015-06-16

**Authors:** Bhanu Malhotra, Anu Keshwani, Harsha Kharkwal

**Affiliations:** ^1^Amity Institute of Biotechnology and Amity Centre for Carbohydrate Research, Amity UniversityNoida, India; ^2^Amity Institute of Phytomedicine and Phytochemistry and Amity Center for Carbohydrate Research, Amity UniversityNoida, India

**Keywords:** antimicrobial food packaging, antimicrobial agents, food safety, shelf life improvement, food spoilage

## Abstract

Nowadays food preservation, quality maintenance, and safety are major growing concerns of the food industry. It is evident that over time consumers’ demand for natural and safe food products with stringent regulations to prevent food-borne infectious diseases. Antimicrobial packaging which is thought to be a subset of active packaging and controlled release packaging is one such promising technology which effectively impregnates the antimicrobial into the food packaging film material and subsequently delivers it over the stipulated period of time to kill the pathogenic microorganisms affecting food products thereby increasing the shelf life to severe folds. This paper presents a picture of the recent research on antimicrobial agents that are aimed at enhancing and improving food quality and safety by reduction of pathogen growth and extension of shelf life, in a form of a comprehensive review. Examination of the available antimicrobial packaging technologies is also presented along with their significant impact on food safety. This article entails various antimicrobial agents for commercial applications, as well as the difference between the use of antimicrobials under laboratory scale and real time applications. Development of resistance amongst microorganisms is considered as a future implication of antimicrobials with an aim to come up with actual efficacies in extension of shelf life as well as reduction in bacterial growth through the upcoming and promising use of antimicrobials in food packaging for the forthcoming research down the line.

## Introduction

Food spoilage is the process of contamination of foods leading to loss of color, texture, and their nutritive value and permits growth of pathogenic microbes, which deteriorates the quality of the product and makes it non-edible. Food contamination can occur with its exposure to the environment while slaughtering, food processing, and packaging. Some traditional food preservation techniques like drying, freezing, heating, fermentation, salting can extend the shelf-life of food products, but recontamination may occur that may render the food unpalatable for the consumers. Antimicrobial packaging system is a novel development which incorporates antimicrobial agent into a polymer film to suppress the activities of targeted microorganisms that are contaminating foods (Sung et al., 2013). Food pathogens endangering consumers’ health can be dealt with the multifunctional bio-based antimicrobial packaging agents that sought to provide enhanced food safety (Sung et al., 2013). The antimicrobial activity can be achieved by adding antimicrobial agents in the packaging system to prevent the growth of microbes by extending the lag period and decreasing live counts of microorganisms by reducing growth rate (Han, 2000). Conventional food packaging aims at shelf life extension, maintenance of quality, and assurance of safety of the food product. However, nowadays food security is a big issue and, therefore, antimicrobial packaging system is specifically designed to control the microorganisms that adversely affect the above three goals.

The girth in the prevention of food contamination by the use of antimicrobial agents is now quickly creating a lot of scientific interest in the world leading to extensive research on them. This review brings about a precise picture of the research on the utilization of various antimicrobial agents for preservation due to microbial spoilage. It also highlights the intelligent active and controlled release packaging technologies that are used to deliver the antimicrobial to the food systems, and elucidates a review over the antimicrobial incorporated food packaging films in the last decade. However, little drawbacks of antimicrobials have also been discussed, which point out the disparity in the laboratory grade results and the real time applications. The future need of developing intelligent packaging technology by selecting the right antimicrobial package and safety regulations have also been considered.

Since the quality, safety, and shelf life of the food product has gained significant attention nowadays, still there are increasing concerns over food spoilage and its ingredient sourcing trouble among consumers (Burt, 2004). Therefore, these concerns demand a need for more effective food quality regulation systems for its protection, preservation, and transport to consumers in a wholesome form. Increase in consumer desire for natural, local, and organic products necessitates efficient food preservation from microbial contamination (De Kruijf et al., 2002; Suppakul et al., 2003; Burt, 2004). Current conventional methods for maintaining food quality over time have not found to satisfy consumers as they demand products extracted from natural sources. Therefore, there is a need in the food industry to explore alternatives to presently used chemicals. Bacterially synthesized antimicrobial peptides known as bacteriocins which are derived from grapefruit extracts and mustard oil have known to be the recent inventions in this field (Chikindas et al., 1997; Kim et al., 2002; Han, 2003; Cha and Chinnan, 2004). Several natural-substances like antimicrobials, antioxidants, have proven out to be effective in laboratory applications, but their effectiveness in real life applications is still challenged by the specific characteristics of the foods and conditions of application.

There is also a requirement for an efficient method for the delivery of antimicrobial substances into food packaging materials. Addition of antimicrobials directly into food; called as formulation, and food wrapping films is regulated by FDA which specifies safety levels of the antimicrobial substances that could be added to food. The addition of antimicrobials instantly in food packaging film formulations often results in instant inhibition of undesired microorganisms. However, the survivor population will continue to grow, as soon as antimicrobials added will get depleted. This is primarily due to complex interactions with the food matrix or by natural degradation with time causing loss of shelf-life (Kester and Fennema, 1986; Ouattara et al., 2000b; Chi-Zhang et al., 2004).

## Antimicrobial Agents

Antimicrobial agents have different activities on different pathogenic microorganisms due to their various diverse physiologies. Antimicrobial agent is integrated either directly into food particle or to the packaging material where it is released over a period of time to maintain the products quality, as well as its safety leading to its extended shelf life. Characterization of microorganisms can be very helpful for the choice of an antimicrobial agent. This can consist of cell wall composition- Gram-negative and Gram-positive, oxygen requirements- aerobes and anaerobes, growth stage- spores and vegetative cells, acid/osmosis resistance, optimal growth temperatures- mesophilic, thermophilic, psychotropic. Some antimicrobial agents inhibit the essential metabolic pathways of microorganisms and some cell wall/membrane structure. Example- lysozyme without the inhibition of metabolic pathways causes the destruction of cell wall while EDTA and lactoferin behave as coupling agents of charged polymers and cationic ions. Antimicrobial agents, therefore, perform two important functions- microbial cidal and microbial static effects. For microbial static effects, the antimicrobial substance has to possess the active function of maintaining the concentration above the minimal inhibitory concentration during the entire storage period or shelf life in order to prevent the growth of target microorganisms. Various antimicrobial substances that have been incorporated into food packaging systems are presented in **Table 1** These commonly include antioxidants, antimicrobials, biotechnology products, natural antimicrobials, antimicrobial polymers, essential oils, etc.

**Table 1:** Numerous antimicrobial agents in food packaging systems.

Antimicrobials	Packaging materials	Foods	Microorganisms
**Organic acids**
a. Benzoic acid	PE LDPE	Tilapia fillets Simulants	Total bacteria Migration test
b. Sorbates	LDPE MC/chitosan Starch/glycerol	Cheese Culture media chicken breast	Yeast, mold Migration test Migration test
c. Sorbic anhydride	PE	Culture media	*Saccharomyces cerevisiae*, molds
**Enzymes**
a. Lysozyme, Nisin, EDTA	SPI, zein	Culture media	*Escherichia coli, Lactobacillus plantarum*
b. Immobilized lysozyme	PVOH, nylon, cellulose acetate	Culture media	Lysozyme activity test
c. Glucose oxidase	Fish	
**Bacteriocins**
a. Nisin	PE HPMC	Beef Culture media	*Brochothrix thermosphacta Staphylococcus aureus*
b. Lauric acid	Zein	Simulants	Migration test
**Fungicides**
a. Benomyl	Ionomer	Culture media	
b. Imazalil	LDPE PE	Bell pepper Cheese	Molds
**Polymers**
a. Chitosan	Chitosan/paper	Strawberry	*E. coli*
b. Chitosan, herb extract	LDPE	Culture media	*E. coli, Lb. plantarum, Fusarium oxysporum, S. cerevisiae*
c. UV/excimer laser irradiated nylon	Nylon	Culture media	*Enterococcus faecalis, S. aureus*
**Natural extract**
a. Grapefruit seed extract	LDPE, nylon	Ground beef	Aerobes, coli-forms
b. Clove extract	LDPE	Culture media	*E. coli, Lb. plantarum, Fusarium oxysporum, S. cerevisiae*
c. Eugenol, cinnamaldehyde	Chitosan	Bologna, ham	Enterobacter, lactic acid bacteria, *Lb. sakei Serratia* sp.
d. Horseradish extract	Paper	Ground beef	*E. coli* s
**Oxygen absorber**
a. Ageless	Sachet	Bread	Moulds
b. BHT	HDPE	Breakfast cereal	
**Gas**
a. Ethanol	Silica gel sachet	Culture media	
**Essential oils**
a. Clove essential oils	Clove, Cyprus, citronella etc	Food packaging films	*Aspergillus niger, Bacillus Coagulans, Bacillus cereus*
**Others**
a. Silver zeolite	LDPE	Culture media	*E. coli, S. aureus, S. cerevisiae, Salmonella typhimurium*
b. Antibiotics	PE	Culture media	*E. coli, S. aureus, S. cerevisiae, Sal. typhimurium, Klebsiella neumoniae*

*Adopted from: Han, 2003; Gómez-Estaca et al., 2010; Pires et al., 2013; Sung et al., 2013. PE, polyethylene; HDPE, high-density polyethylene; LDPE, low-density polyethylene; HPMC, hydroxypropyl methylcellulose; MC, methylcellulose.*

## Packaging Technologies

### Active Packaging

Active antimicrobial packaging is used to actively modify the internal environment by continuous interaction with the food over the stipulated shelf-life. Active packaging can be defined as a system that modifies the environment inside the food package thereby altering the state of the packaged food system and its headspace to enhance its quality by extension of shelf-life, enhancement of sensory qualities, and maintenance of microbial safety (Debeaufort et al., 2000; Quintavalla and Vicini, 2002; Han, 2003; Suppakul et al., 2003)^.^ The increasing popularity of active packaging is due to the desire for high-quality safe and natural products amongst consumers (Han, 2003; Cha and Chinnan, 2004; Ozdemir and Floros, 2004). In this technique, the packaging materials are actively interacting with the food product. Some of the active packaging systems include O_2_ or CO_2_ scavengers, ethylene and moisture absorption systems, CO_2_ or ethanol emitting systems, and antimicrobials antioxidants releasing or containing systems (De Kruijf et al., 2002; Han, 2003; Cha and Chinnan, 2004; Ozdemir and Floros, 2004). Scavenging systems which have now been commercialized in Japan and USA. can absorb deleterious compounds from the food surface or from the headspace and emitting systems can release certain compounds which act at the surface of the food or within its headspace, **Table 2** enlists various active packaging technologies and their use in food packaging applications (Quintavalla and Vicini, 2002; Han, 2003). Materials like ascorbic acid, photo-sensitive dyes, iron powder, are packed to scavenge oxygen and thereby to prevent the growth of aerobic bacterias and molds (Floros et al., 1997).

**Table 2:** Various active packaging technologies enlisting their potential food applications.

Active packaging techniques	Potential food applications
O_2_ Scavenging systems	Roasted nuts, chocolates, meat sausages, chicken salami, cereals, cookies, beer, bread
CO_2_ Scavenging systems	Coffee, poultry products
CO_2_ Emitters	Nuts, cake, potato chips
Antimicrobial release systems	Bread, cheese, meat, cakes
Antioxidant release systems	Cereals, wine
Ethanol emitters	Cheese, fish, bakery products
C_2_H_4_ Emitters	Minimally processed foods
Moisture scavenging systems	All dry fruits and nuts, fish, bread, sea food, meat

*Adopted from: Suppakul et al., 2003; Gibis and Rieblinger, 2011; López et al., 2012.*

### Controlled Release Packaging

Controlled release packaging, which is a relatively new technology called controlled release packaging is one of the sophisticated form of active packaging which centers its focus on the releasing systems. It can be an active system which utilizes packaging as a delivery vehicle to efficiently bring the actives in specifically controlled rates over prolonged periods to the food product to further improve its quality and safety (Lacoste et al., 2005). Control release packaging regulates the concentration of the antimicrobial agents in food at a particular targeted level which is effective in deteriorating microbial growth kinetics and making it safe for consumption. Controlled release packaging is also known as time-release or slow-release packaging of active substances and it has been in existence for many years as a marketable method of utilizing drugs like antibiotics and antimicrobials for various food packaging applications. It can also be used to release vitamins, prescription medications, and antioxidants into varieties of environments. The concept of controlled release packaging is still being explored for food packaging. According to extensive research findings it has been postulated that the key parameters of mathematical modeling play a significant role in the controlled release of an antimicrobial agent from the polymeric network (Mastromatteo et al., 2010). Controlled release of components like enzymes, flavors, sweeteners, and other food preservatives has been accomplished using encapsulation (Karel, 1987).

A natural antimicrobial known is lysozyme inhibits lactic acid bacteria causing wine malolactic fermentation are incorporated in PVOH films. The degree of crosslinking of PVOH films helps to maintain the release rates of the antimicrobials to provide an effective inhibition (Buonocore et al., 2003). Similarly, potassium sorbate’s antibacterial and antimycotic effect when added to HDPE and LDPE films has been studied on American cheeses. Released sorbate from HDPE films was found to be effective in enhancing cheese storage for 5 months at room temperature (Szente and Szejtli, 2004).

## Applications of Antimicrobials

### Food Products and Food Packaging Films

From an instance, there is a growing interest in edible film coatings due to environmental concerns of synthetic polymer based packaging materials. Edible packaging films incorporating antimicrobial coatings have offered an innovation within the biodegradable active packaging concept. They have been used to reduce and inhibit the growth of microorganisms on the surface food products. A polymer-based film solution coating has proved to be the most desirable method in terms of stability of attaching a bacteriocin to a plastic packaging film (An et al., 2000). Low-density polyethylene (LDPE) packaging film was coated with nisin using derivatives of natural polymers like methylcellulose (MC) and hydroxypropyl methylcellulose (HPMC) as a carrier. Nisin was found suppressing the growth of *Staphylococcus aureus* and *Listeria Monocytogenes* (Cooksey, 2000). It was also suggested that the combination of packaging material nisin used along with nisin containing foods will provide a means of preventing *L. monocytogenes* growth; with a conclusion that the antimicrobial effectiveness of nisin depends very strongly on the mode of delivery.

Incorporating bacteriocins into food packaging films to control spoilage of food pathogenic microorganisms has been an area of research for the last decades. Antimicrobial packaging in films prevents microbial growth on the food surface by direct contact of the packaging material with the surface of foods. This is the reason for which the antimicrobial packaging film should ensure its contact with food surface leading to diffusion of bacteriocins to its surface. The controlled release of bacteriocins from food packaging film toward the food surface has a good advantage over dipping, spraying foods with bacteriocins. Antimicrobial activity is lost or decreased because of inactivation of the bacteriocins by components of food or their considerable dilution below active concentration after their migration into the foods (Appendini and Hotchkiss, 2002). Two procedure shave been commonly used to make packaging films with bacteriocins. Two packaging methods, heat-press, and casting, were used to deliver nisin into films made from soy protein and corn zein protein. Both the procedures produced excellent films by inhibiting the growth of the microbe *L. plantarum*. When the same levels of nisin were incorporated cast films exhibited larger inhibitory zones than the heat-press films. It was also shown that when EDTA was incorporated into the films increased the inhibitory effect of nisin against the bacteria *Escherichia coli*.

Edible cellulosic films made with nisin formulations using HPMC were also produced (Coma et al., 2001). Controlled release application in case of bacteriocins offers a treatment strategy for resistant bacterial strain (Benoit et al., 1998; Chi-Zhang et al., 2004). Research has elucidated that the instantaneous release of nisin can help inhibit microbial cell growth and consequently the survivors will undergo mutations developing resistance to nisin. Also merely releasing nisin from packaging without adding directly to the formulations was not capable of reducing microbial cell counts. A combination of these two resulted both in reduced cell counts as well as a lack of mutation but instead the cells regained their sensitivity to nisin following one transfer passage through nisin-free medium (Chi-Zhang et al., 2004). Nisin- coated polymeric films like PVC, nylon, and linear low- density polyethylene (LLDPE), have found out to be effective in inhibiting *Salmonella typhimurium* particularly on fresh broiler drumstick skin (Natrajan and Sheldon, 2000). LDPE films which were coated with a mixture of polyamide resin prepared in *n*-propanol along with a bacteriocin solution showed an antimicrobial activity against *Micrococcus flavus*. It was also showed that the efficiency of Nisin coated over LDPE film caused inhibition of *M. luteus* ATCC 10240 and the microbiota of raw milk during storage (Mauriello et al., 2005).

According to a relationship between polymer structure and the transport of active molecules, has been elucidated. γ-irradiation along with heat have shown to produce cross-linking within protein molecules leading to improved physical, functional, and chemical properties of edible films. Modification of the structure was found to increase the capacity of edible films to modulate the release of immobilized active compounds. LLDPE films hydrophobic properties rejected hydrophilic nisin preparations to a greater extent than the other films by causing coalescence of the droplets (Papadokostaki et al., 1997).

Polyanhydrides are biodegradable polymers which have gained popularity in biomedical applications like drug delivery, tissue scaffolds, and implant coatings. They contain hydrophobic compounds which are bound by hydrolytically labile anhydride bonds, and the rate of their degradation can be controlled by altering polymer composition (Schmeltzer et al., 2005). Anhydrides added to packaging films made of natural polymers have shown a potential antimicrobial activity^5^. These packaging films can act as prodrugs, breaking down to release the antimicrobial agent in a controlled manner in the desired area. Poly anhydride esters or PAE polymers can controllably used in combination with other active substances to enhance food quality and safety (Bryers et al., 2006; Rosenberg et al., 2008). These films have an upcoming potential in the food industry. Antimicrobial agents are used as additives in films and food products successfully for many years. The directly incorporating antimicrobial additives in food packaging films based on natural and synthetic polymers is a convenient methodology by which activity of antimicrobial substances can be realized. In the past literature provides evidence of some of these additives affectivity as indirect food additives incorporated in food packaging materials. Several antimicrobial agents have been checked and tested for antimicrobial packaging using this technique. However, their use is not a substitute for good sanitation practices, but it enhances the safety of food as an additional hurdle to the growth of pathogenic microorganisms.

Milk protein based film containing 1.0 % (w/v) pimento, 1.0 % (w/v) oregano, or 1.0 % oregano – pimento (1:1) essentials oils showed antimicrobial and antioxidant effects for the preservation of beef muscle by controlling the growth of pathogenic bacteria and thereby increasing their shelf life during storage at 4°C. They also showed that film which contained oregano was most effective against *Pseudomonas* sp and *E. coli* O157: H7, whereas film which contained pimento oil, was least effective against these above discussed bacteria. Films having oregano extracts, showed 0.95 log reduction of *Pseudomonas* sp at the end of storage, as compared to the samples which did not have oregano extracts. The 1.12 log reduction of *E. coli* O157: H7 level was seen in samples having a coating of oregano (Oussalah et al., 2004).

By incorporating of 1.0% w/w potassium sorbate in LDPE (about 0.4-mm thick) it was found that potassium sorbate lowered the maximum growth rate of yeast, by lengthening the lag period before yeast mold growth became apparent. Potassium sorbate shows antimicrobial activity against yeast molds and many bacterial species (Han and Floros, 1997). Whereas contradictory results were obtained on LDPE (0.05-μm thick) containing 1.0 % w/w sorbic acid was shown to be unable to suppress yeast mold growth when was in contact with inoculated media. Because of the incompatibility of the polar salt with the non-polar, limited migration of potassium sorbate into water and cheese cubes was seen in LDPE films occurs (Weng and Hotchkiss, 1993). Research confirmed that ethylene vinyl alcohol linear LDPE (EVA/LLDPE) film (about 70-mm thick) incorporated with 5.0% w/w potassium sorbate is unable to inhibit microorganisms growth on cheese and could not extend its shelf life (Devlieghere et al., 2000; Türe et al., 2012).

Chitosan films made from dilute acetic acid solutions were found to inhibit the growth of *Rhodotorula rubra* and *Penicillium notatum* if the film preparation was applied directly to the colony-forming organism (Chen et al., 1996). Because of the solubility of chitosan only in slightly acidic solutions, production of films containing the salt of an organic acid like benzoic acid, sorbic acid showed antimicrobial activity. However, the interaction between the film-forming material and the antimicrobial agent and is shown to affect the casting process, as well as the release of the antimicrobial agent and the mechanical properties of the film. Starch based films containing chitosan was developed in 2009 (Vásconez et al., 2009; Leceta et al., 2013). The effect of antimicrobial activity of chitosan was observed by zone inhibition technique on agar. (Zhong et al., 2011). A number of studies on chitosan films by evaporating dilute acids on the antimicrobial characteristics of films made from chitosan have been performed earlier (Ouattara et al., 2000a; Coma et al., 2002; Rivero et al., 2013). The antimicrobial efficiency of chitosan along with HPMC as films, with stearic acid preparations produced by chemically modifying crosslinking obtained by citric acid, were evaluated recently. Film preparations without stearic acid inhibited the growth of *L. monocytogenes* completely (Möller et al., 2004).

Edible films incorporating garlic oil with chitosan were prepared using potassium sorbate as a conventional food preservative with bacteriocin nisin at various concentrations, showed effective antimicrobial activity against *Bacillus cereus, E. coli*, *Salmonella typhimurium*, *Staphylococcus aureus*, and *L. monocytogenes*. Garlic oil could be added to the preparation only with the type of foods where its flavor is not a problem (Pranoto et al., 2005). Use of chitosan-based films to inhibit *L. monocytogenes* specifically and chlorine dioxide sachets for the reduction of *Salmonella* in modified atmosphere packaged fresh chicken breast were also researched (Cooksey, 2005).

## Antimicrobial Packaging: Lab Scale and Real-Time

The growing concern with antimicrobial food packaging is the extrapolation of laboratory results into that of the real world. Often, laboratory grade tests are performed with food simulants which are supposed to be far less complex than actual food systems^9^. In real food system foods have more salt contents, lower water activity, nutrients, and fats or proteins which are found to interact with the antimicrobials (Walters et al., 2003; Grower et al., 2005; Mauriello et al., 2005). Also, in addition, the conditions in which the food products are stored and transported have shown a considerable effect on their characteristics. Moisture content and temperature and have shown a great effect on release rates of compounds along with their effectiveness (Szente and Szejtli, 2004). Release rate being a significant parameter because it determines the amount of the antimicrobial compound emerging from the packaging and how long it takes to saturate the area. When we employ simple diffusion of antimicrobials they are found to diffuse across the gradient, but as the surface of the food, or the headspace becomes saturated, their release is lowered, and stopped (Han, 2003).

Due to this many substances undergo an initial burst effect, thereby releasing a lot of antimicrobials all at once and ceasing their release until all of it is consumed. The release of the active substance is governed by swelling and water uptake of the film not solely by diffusion. Various microbiological, analytical, and chemical tests can determine where the antimicrobial agent goes and in how much quantities (Chi-Zhang et al., 2004; Szente and Szejtli, 2004). However, achieving the same results in real food system is thought to be far more challenging. While testing real foods with antimicrobials it is reported, that antimicrobial packaging system is less effective than it was under laboratory scale experiments (Kim et al., 2002; Duan et al., 2007). Tests with essential oils as antimicrobial agents have revealed that considerably much higher levels of them are needed to achieve antimicrobial effect in foods as compared to lab scale in milk and cheese applications. The suggested explanations for this phenomenon are higher organic acid and trace metal content, greater availability of nutrients for cellular repair, and interactions with compounds in the food that may interact with or inactivate the active substance (Burt, 2004).

Packaging incorporating triclosan was investigated as an antimicrobial in food applications. It was found that no triclosan got released in pure water and in 10% ethanol solution only less than 2% was released with which approximately 2-log reduction of *Enterococcus faecalis* was achieved with this amount. While this reduction was found to be impressive for such a small amount of antimicrobial, because of the high minimum inhibitory concentration of triclosan, it could not be preferred to prevent illness in humans (Chung et al., 2003). In addition to this another study using triclosan-containing packaging films revealed lower inhibition in laboratory conditions but none when checked to chicken breasts vacuum-packed and stored at 7°C in real application (Walters et al., 2003). Therefore, it was concluded that due to correlation between resistance to triclosan and resistance to other antimicrobials the widespread use of triclosan should not be encouraged in food systems. Perhaps the use of triclosan in significant low amounts should be effective in combination with natural antimicrobials to the kill the bacterial system providing a preservation system with multiple modes of action and preventing resistance from becoming more widespread (Suppakul et al., 2003).

Due to the lack of proper study design, including measurements of essential parameters including amount of substance released and amount of substance retained, pH, and growth kinetics it was seen that nisin release from cellulose-based packaging films showed failure of nisin to inhibit *L. monocytogenes* for neutralization of the bacteriocin by peptone waters pH used as a food stimulant. But pH was not measured over time, and no data was recorded revealed that no zones of inhibition were seen at first 30 min and 4 days but seen at 8th day. From this it was concluded that the nisin released in the first 30 min was neutralized by 24 h, and after it took time from 4 to 8 days for the remaining amount of nisin to be released to act against the pathogenic microorganism to overcome the pH neutralization. But there were no data to confirm this assumption besides the only fact that inhibition occurred at the 8th day while at 4 days no inhibition was seen. This lag in release can lead bacteria not only to overcome the stress of the antimicrobial agent used against it but also for the survivor population to develop resistance (Grower et al., 2005).

The study along the same lines evaluated LDPE films incorporating nisin and lactocin to extend the shelf life of real food systems beyond their current limits for fresh oyster and ground beef packaging. Unfortunately, the laboratory results which were very promising did not translate into real time consideration for an extension of the shelf-life in this case, exhibiting less than 2-log reduction in total aerobic and coliform counts at 10∞C for both ground beef ad oysters (Kim et al., 2002). Therefore more and more research is the need to further prevent cell growth through higher concentrations with combinations of antimicrobials.

## Future Considerations

Safety evaluations for active antimicrobial packaging with a potential to enhance food safety along with preventing the formation of resistant strains of microorganisms is a prerequisite for future. For this testing for the occurrence of resistance in the survivor population of microorganisms is required for safety evaluations of an antimicrobial agent to be used as packaging materials. The study involving cellulose casings for frankfurters incorporating nisin showed the antimicrobial nisin resistance developed in cells from prolonged exposure to the antimicrobial but was never pursued in the upcoming research. After 90 days which is sought to be typical shelf-life for refrigerated frankfurters, *L. monocytogenes* reached the same cell density in control as well as in nisin-coated samples. There was no indication testing done to determine if the survivors had developed the nisin resistance following the treatment. If actually the cells had become resistant, then theoretically using the antimicrobial packaging could be a worse scenario for food safety (Mathiowitz et al., 1997).

Active packaging technology passively protects food items, by not only inhibiting the pathogenic growth, but by also providing an extended shelf life combating a variety of environmental factors. Since the nano material antimicrobial agents possess a higher surface area-to-volume ratio in comparison to their normal counterparts, they are considerably more efficient against food pathogens (de Azeredo, 2013). Various antimicrobials based nano composite food packaging films incorporated with antimicrobial agents have shown enhanced thermal, physicochemical, mechanical, and optical properties. The recent researches on nano based films with principle antimicrobials are being studied nowadays to improve the efficacy of delivery of such agents (Tunç and Duman, 2011).

Although the use of many materials in active packaging systems are safe for use in food products on their own, but the mere act of incorporating them into a new packaging system is subject to the regulatory rules. Some of the essential oil components used as flavorings in the EU and have GRAS recognition in the U.S., while others are specifically prohibited for toxicological reasons due to the fact that some can cause irritation due to cytotoxic effects while others can be allergens. The use of menthol, thymol, and eugenol in the treatment of root canal, has shown to cause irritation of mouth tissues due to membrane lysis and tissue penetration for example. While *in vivo* thymol, cinnamaldehyde, carvone, and carvacrol show negligible effects but *in vitro* they show moderate toxic effects at the cellular level. In addition, organoleptic changes, i.e., change in color texture and appearance of food product may occur due to the release of some antimicrobials like essential oils (Burt, 2004; Tongnuanchan et al., 2012). Oregano oil of concentration 0.05% v/w over cod filets exhibited a different pleasant flavor which was found to decrease gradually on storage. Oregano oil and thyme over whole Asian sea bass produced a herbal odor, which became more pronounced over storage. Carvacrol produces a pungent aroma on fish (Kim et al., 2002). Comparing the inherent imaginable potential of antimicrobials agents in food products and its reality it is pretty obvious that though these agents have a realm of possibility for packaging industry for inhibiting microbial growth and extending the shelf life of foods, a cutting edge research is still a requirement seeing the above-mentioned limitations.

## Conclusion

With the evident differences between lab scale and real food packaging applications, a slow sustained delivery of antimicrobials is a very important factor that can mean the difference between life and death. It is crucial to evaluate a microbe for which antimicrobial is to be targeted. When microbes with very short lag periods are a reason of food spoilage selecting a polymer which releases the antimicrobial very slowly over a period of time will not prove out to be effective. Therefore, it is essential to select the right package of the antimicrobial agent and environmental condition for a particular food product. Although it might happen if the antimicrobial is too compatible it may not get released or incompatible that will be released within minutes. Proper care should be taken while selecting and incorporating the antimicrobial into the food packaging film by evaluating all the implications of food safety and harsh reality of microbial resistance. Thus based on the research work carried out by various scientist around the globe it is imperative that we establish the use of a multidisciplinary approach by bringing together experts from all the fields of biotechnology particularly microbiology, food technology, engineering, and material science to create a promising withstanding future of antimicrobials in food packaging industry.

## Conflict of Interest Statement

The authors declare that the research was conducted in the absence of any commercial or financial relationships that could be construed as a potential conflict of interest.
